# Integrating the One Health approach into school curricula: perceptions and educational needs of teachers in mainland France and New Caledonia

**DOI:** 10.3389/fpubh.2026.1822865

**Published:** 2026-04-24

**Authors:** Laure Hermet, Pauline Coraci, Pia Touboul-Lundgren

**Affiliations:** 1 Department of Public Health, Nice University Hospital, Nice, France; 2 UR2CA – RESPECT, Université Côte d’Azur, Nice, France

**Keywords:** antimicrobial resistance, health literacy, implementation barriers, One Health, qualitative research, school health education, vector-borne diseases

## Abstract

**Introduction:**

The “One Health” approach highlights the interdependence between human, animal, and environmental health, yet its integration into school curricula has been scarcely documented. This study explores teachers’ perceptions and educational needs regarding its integration in mainland France and New Caledonia, within the framework of the e-Bug program.

**Methods:**

A qualitative study was conducted based on four focus groups involving 25 primary and secondary school teachers (January–February 2025). Discussions were transcribed and analyzed using an inductive thematic approach with NVivo software.

**Results:**

Four main themes emerged: limited familiarity with the term “One Health,” despite an intuitive understanding of the links between environmental, animal, and human health; perceived relevance varying across territorial contexts, with a stronger sense of risk in New Caledonia; strong expectations for active, progressive, interdisciplinary, and ready-to-use resources; and implementation barriers related to time constraints, limited scientific confidence, concerns about inducing anxiety among younger pupils, and material conditions.

**Conclusion:**

Making the One Health concept explicit, aligning it with school curricula, and providing contextualized and reassuring resources for teachers appear essential to facilitate its integration into school settings.

## 1 Introduction

Over the past decades, health crises have multiplied worldwide. While major pandemics have punctuated human history, the second half of the twentieth century and the beginning of the twenty-first century have been characterized by an acceleration in the emergence and re-emergence of infectious diseases (1, 2). Epidemics related to HIV (Human Immunodeficiency Virus), severe acute respiratory syndrome (SARS), pandemic influenza, Ebola, and more recently COVID-19 have illustrated both the increasing frequency of epidemic events and their rapid global spread in an interconnected world. This dynamic unfolds within a context of profound environmental transformations, including intensified international trade, rapid urbanization, land-use change, biodiversity loss, and climate change (3).

In this increasingly complex infectious risk landscape, strictly biomedical approaches appear insufficient. Interactions between human activities, ecological dynamics, and animal health now play a central role in the mechanisms underlying pathogen emergence. Within this context, the “One Health” approach has emerged as a structuring conceptual framework. It is grounded in the recognition of the close interdependencies between human health, animal health, and ecosystem health, and promotes an integrated understanding of contemporary health challenges (4, 5).

Antimicrobial resistance (AMR) provides an emblematic example of this interdependence. Considered by the World Health Organization (WHO) as one of the leading threats to global public health (6), the emergence and spread of resistant bacteria result from complex interactions among human, veterinary, and agricultural uses of antibiotics, as well as from the dissemination of pharmaceutical residues and resistant bacteria in soils, water systems, and ecosystems. The environmental impact of biocides and antimicrobials further contributes to the selection of resistance, highlighting the systemic nature of the phenomenon. AMR therefore cannot be fully understood or effectively prevented without an integrated approach linking human, animal, and environmental health. In Europe, infections caused by antibiotic-resistant bacteria are responsible for tens of thousands of deaths each year, underscoring the magnitude of the challenge (7).

Vector-borne diseases offer another concrete illustration of these dynamics. In mainland France, dengue and chikungunya, once considered imported diseases, now give rise to autochthonous cases and localized outbreaks, particularly along the Mediterranean coast, where a significant epidemic surge was observed around Antibes in 2025 (8). The sustained establishment of mosquito vectors, facilitated by climatic conditions and human mobility, exemplifies the interconnections between environment, fauna, climate, and human health. These developments render the One Health framework tangible at the national level in France.

French overseas territories provide complementary insights. In New Caledonia, long-standing experience in the control of vector-borne diseases, including innovative programs such as the introduction of *Wolbachia*-infected mosquitoes (9, 10), reflects adaptive responses to health challenges associated with island ecosystems. At the same time, the recent increase in leptospirosis cases (11, 12), a zoonosis influenced by environmental and social conditions as well as human and animal interactions, highlights the complexity and persistence of infectious risks in these settings.

Together, these phenomena illustrate the interconnectedness between environmental changes, microbial vectors, animal hosts, and human health, reinforcing the need to frame prevention strategies within a One Health perspective.

Although the One Health approach is now widely mobilized within scientific and institutional spheres, its operationalization within educational systems remains poorly documented. Yet schools represent a strategic setting for fostering early understanding of the links between environment and health, strengthening critical thinking skills, and encouraging preventive behaviors (13–15). Health and environmental education are already embedded within French curricula, particularly through life and earth sciences, geography, and civic education. However, the explicit integration of a structuring framework such as One Health remains limited.

The international e-Bug programme (16, 17) aligns with this primary prevention perspective by developing educational resources for teaching primary and secondary school students about infections, their transmission, prevention strategies, and antimicrobial resistance. In response to evolving global health challenges, these resources have increasingly incorporated the One Health approach to promote a systemic understanding of health issues and to support the teaching of this complex topic in classroom settings.

Exploring how teachers perceive and envision the integration of the One Health approach makes it possible to identify both facilitators and barriers to its pedagogical implementation, while taking territorial specificities into account. This perspective supports the development of educational resources that are context-sensitive, scientifically robust, and aligned with curricular requirements.

Within this framework, the present qualitative study aims to analyze the perceptions, representations, and pedagogical needs of primary and secondary school teachers in mainland France and New Caledonia regarding the development of curriculum-aligned educational resources on the One Health approach.

## 2 Materials and methods

### 2.1 Study design

An exploratory qualitative study was conducted to identify teachers’ perceptions, understandings, and pedagogical needs regarding the development of educational resources on the “One Health” approach and their integration into school curricula. This study design was considered the most appropriate to capture teachers’ experiences and perspectives on these complex issues, and to allow for the emergence of themes not anticipated *a priori* (18). Qualitative methods are particularly suited to exploring complex, context-dependent phenomena and to capturing participants’ subjective experiences and meanings (19–21). Focus groups were chosen as they enable interaction between participants, facilitating the emergence of collective views, shared experiences, and diverse perspectives. The study was based on four focus groups (FGs) conducted between January and February 2025 with primary and secondary school teachers in mainland France and New Caledonia. Focus groups were conducted until data saturation was reached, defined as the point at which no new relevant themes emerged from the discussions.

### 2.2 Participants and recruitment

Teachers were recruited through the dissemination of an invitation letter via regional education inspectors, as well as through the French Association of Biology and Geology Teachers (Association des Professeurs de Biologie et Géologie, APBG). A non-probabilistic purposive sampling strategy was used to recruit participants, aiming to capture a diversity of perspectives across educational levels and territorial contexts. The letter outlined the study objectives, participation procedures, and included a detailed information sheet. Teachers who were interested contacted the research team directly by email. An informed consent form was then sent to them. Participation in the focus groups was conditional upon the signed return of this form. The dates and times of the focus groups were subsequently scheduled according to participants’ availability. Inclusion criteria were being a primary or secondary school teacher currently teaching in mainland France or New Caledonia, and willingness to participate in a focus group discussion. Exclusion criteria included not currently being in active teaching practice or not working within the French educational context.

The selection of mainland France and New Caledonia was guided by their contrasting epidemiological and environmental contexts, particularly regarding vector-borne diseases. In mainland France, these diseases are considered emerging, whereas in New Caledonia they represent long-standing public health issues, allowing exploration of how different levels of exposure to health risks may influence teachers’ perceptions and practices.

**Figure 1:**
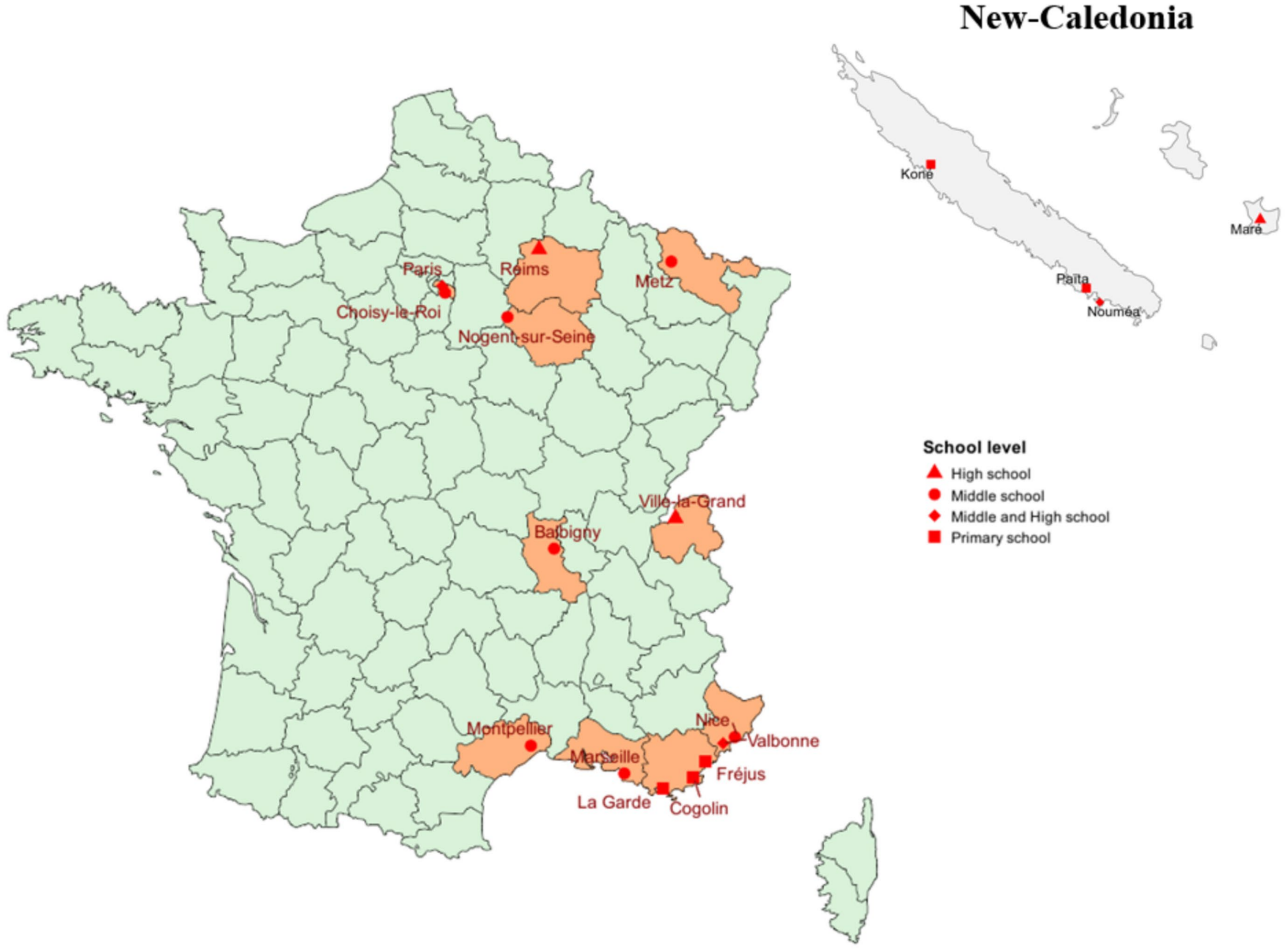
Geographical distribution of teachers participating in the focus groups.

A total of four focus groups were conducted, involving 25 participants. These included upper secondary school (*n =* 6), lower secondary school (*n =* 7), primary school (*n =* 4), and New Caledonia (*n =* 8; including primary, lower secondary, and upper secondary levels). The sample was predominantly composed of female teachers (*n =* 22), reflecting the gender distribution of the teaching profession (Table 1). Participants were drawn from 14 cities in mainland France and 4 municipalities in New Caledonia, representing urban, peri-urban, rural, and island contexts (Figure 1). The mainland sample predominantly consisted of science teachers, whereas the New Caledonian group also included primary generalist teachers, pedagogical advisors, and one education inspector, thereby ensuring a diversity of institutional and territorial perspectives. In mainland France, focus groups were organized by educational level (primary, lower secondary, and upper secondary) to facilitate discussions among teachers sharing similar pedagogical contexts and to identify level-specific issues. In New Caledonia, participants were grouped into a single focus group. This decision was driven by organizational constraints, including the time difference, as well as a smaller number of participants and uneven representation across educational levels. To structure the discussion, questions were addressed sequentially according to educational levels, allowing participants to contribute based on their teaching context. The New Caledonian group included teachers, one education inspector (a former secondary school teacher), and two pedagogical advisors. Teachers mainly responded to questions related to classroom practices, while pedagogical advisors contributed to curriculum-related aspects, providing complementary perspectives on implementation conditions.

**Table 1:** Characteristics of participating teachers (*n =* 25).

Gender	Region/Territory	Teaching level	Subject taught	Focus group
F	Val-de-Marne	Middle school	Science	Middle school
F	Alpes-Maritimes	Middle school	Science	Middle school
F	Alpes-Maritimes	Middle and High school	Science	High school
M	Moselle	Middle school	Science	Middle school
F	Marne	High school	Science	High school
F	Marne	Middle school	Science	Middle school
F	Loire	Middle school	Science	Middle school
F	Hérault	Middle school	Science	Middle school
F	Haute-Savoie	High school and University	Science	High school
F	Paris	Middle and High school	Science	High school
F	Bouches-du-Rhône	Middle school and University	Science	Middle school
F	Var	Primary school	All subjects	Primary school
F	Var	Primary school	All subjects	Primary school
F	Var	Primary school	All subjects	Primary school
F	Var	Primary school	All subjects	Primary school
F	New Caledonia	Primary school	All subjects	New Caledonia
F	New Caledonia	Primary school	All subjects	New Caledonia
F	New Caledonia	Primary school	All subjects	New Caledonia
F	New Caledonia	Primary school	All subjects	New Caledonia
M	New Caledonia	Middle school	Science	New Caledonia
F	New Caledonia	Primary school	All subjects	New Caledonia
F	New Caledonia	Primary school	All subjects	New Caledonia
M	New Caledonia	Middle and High school	Science	New Caledonia
F	Alpes-Maritimes	Middle and High school	Science	High school
F	Alpes-Maritimes	Middle school	Science	High school

### 2.3 Data collection

The focus groups were conducted via videoconference using Microsoft Teams and lasted between 60 and 90 min, depending on the group. A semi-structured interview guide was used to explore participants’ prior knowledge of vector-borne infections and the One Health approach, their understandings of the links between human, animal, and environmental health, the possibilities for curricular integration, their needs in terms of educational resources, and the perceived barriers and facilitators to teaching these topics. The full interview guide is provided in Supplementary material 1. Discussions were audio-recorded with participants’ consent and subsequently transcribed verbatim into Word documents for analysis.

### 2.4 Data analysis

An inductive thematic analysis was conducted based on the verbatim transcripts, following the approach described *“Using thematic analysis in psychology”* (22). Thematic saturation was considered achieved when no new meaningful themes emerged from the final focus groups. Saturation was assessed through ongoing comparison of codes and themes across focus groups, ensuring that no new categories or significant insights emerged despite the diversity of participants and contexts. This process was applied across all focus groups, allowing comparison between educational levels and territories. The data were imported into NVivo software (QSR International) to enable systematic coding of the corpus. Initial coding was performed by one researcher using an inductive approach, allowing themes to emerge directly from the data without a predefined coding framework. The identified categories and themes were subsequently discussed with two additional members of the research team, including a researcher experienced in qualitative methods, in order to assess their relevance and coherence, refine their definitions, minimize interpretative bias, and strengthen the internal validity of the analysis. This iterative process contributed to enhancing the credibility and trustworthiness of the findings (23). Reflexivity was considered throughout the analytical process, with regular discussions within the research team to reflect on interpretations and potential biases. The final themes were organized into cross-cutting axes, integrating specificities related to educational levels and territorial contexts.

### 2.5 Ethical considerations

Participants received detailed information regarding the study objectives and procedures and provided written informed consent prior to participation. Data were anonymized during transcription and analysis. The study was conducted in accordance with the French Reference Methodology MR-004 governing research involving personal data and was registered on the Health Data Hub platform under number 23258975, March 25 2025.

## 3 Results

Twenty-five teachers participated in the focus groups; their characteristics are detailed in Table 1.

The analysis identified four main thematic axes regarding the integration of the One Health approach into school curricula and the development of dedicated educational resources: representations and understandings of the One Health concept; territorial embeddedness and contextualization of health issues; pedagogical expectations and resource structuring; implementation conditions, including both facilitators and barriers. A cross-analysis of the findings across territories and educational levels is integrated throughout the results and further synthesized in Section 3.5, providing a structured comparison of key similarities and differences. A synthesis table summarizing the distribution of themes across territories and educational levels is provided in Table 2 to enhance analytical transparency. Across focus groups, a high level of convergence was observed in participants’ views, with teachers generally expressing similar perspectives both within and across educational levels. Variations were mainly related to territorial context and, to a lesser extent, to differences between primary and secondary education, as reflected in the thematic analysis.

**Table 2:** Cross-analysis of themes across territories and educational levels.

Theme	Primary	Lower secondary	Upper secondary	New Caledonia
One Health understanding	Intuitive understanding with limited use of terminology	Similar patterns with intuitive understanding and limited explicit framing	More structured understanding, although not explicitly framed as One Health	Intuitive understanding combined with experiential knowledge
Perception of risk	Perceived as low and distant from everyday experience	Perceived as moderate, with some emerging awareness	Mainly linked to climate change and global environmental issues	Perceived as high, concrete, and directly experienced
Pedagogical expectations	Preference for play-based, simple, and highly visual approaches	Preference for active and structured learning approaches	Emphasis on scientific data, critical thinking, and interdisciplinarity	Preference for active and contextualized approaches linked to local realities
Facilitators	Strong preference for ready-to-use resources providing reassurance and guidance	Similar expectations with emphasis on usability and structure	Similar expectations with additional focus on curriculum alignment	Similar expectations with strong emphasis on local relevance and applicability
Barriers	Concerns about student anxiety and lack of time	Time constraints and lack of adapted resources	Complexity of content and limited time	Lack of knowledge and need for contextualized resources

### 3.1 Theme 1—limited familiarity with the concept but intuitive adherence to its principles

#### 3.1.1 Limited familiarity with the term “One Health”

Most teachers reported that they were not formally familiar with the term “One Health,” regardless of educational level or territorial context.

*“In France, no. A general idea, but in France, no”* (teacher 1, primary school).

*“For me, it is completely new”* (teacher 1, New Caledonia).

Even among secondary school science teachers, the concept was not explicitly mobilized as a structuring framework. This limited terminological familiarity contrasts with teachers’ ability to spontaneously mobilize systemic reasoning. It suggests that the One Health concept has not yet been integrated as an explicit reference within professional teaching culture, although its underlying principles are partially present in certain existing practices (education for sustainable development, climate, biodiversity).

#### 3.1.2 Spontaneous understanding of interdependencies

Despite limited familiarity with the term, several teachers intuitively expressed the idea of interconnectedness between the environment, animals, and human health.

*“The disease, it comes at the end of the chain”* (teacher 1, primary school).

*“If we destroy the environment, inevitably it has consequences for us”* (teacher 6, lower secondary school).

*“Everything is connected, the climate, the animals, the humans”* (teacher 3, New Caledonia).

These statements reflect an implicit representation in which disease is perceived as the consequence of ecological imbalance. Teachers view health as the outcome of complex interactions. However, this understanding remains intuitive and is not structured by an explicit conceptual framework. The absence of formal conceptualization may limit the coherence and progressive structuring of teaching on these topics.

This pattern was observed consistently across both territories and educational levels, suggesting that limited familiarity with the One Health terminology is a widespread phenomenon. However, secondary school teachers, particularly those teaching science subjects, tended to express more elaborated reasoning about these interconnections, highlighting variations in depth of understanding rather than in conceptual presence.

### 3.2 Theme 2—territorial contextualization: differentiated proximity to risks

#### 3.2.1 In mainland France: an issue perceived as emerging and distant

In mainland France, vector-borne infections were rarely taught or addressed in depth in the classroom.

*“These are not topics that you already address a little in class? - No”* (teachers 2 and 3, primary school).

*“It is rather seen as exotic diseases”* (teacher 5, upper secondary school).

Several teachers associated these issues with climate change, but in an indirect manner:

*“We talk about it when we cover climate change, but not specifically”* (teacher 2, lower secondary school).

The potentially anxiety-inducing dimension was also mentioned:

*“We really need to work on this anxiety-inducing aspect”* (teacher 1, primary school).

In mainland France, vector-borne infections remain associated with a geographical elsewhere (“*elsewhere*,” “*exotic*”), despite the increase in autochthonous cases. This perceived distance may constitute a barrier to their integration into teaching. Framing the issue through environmental dynamics appears to serve as a strategy for legitimizing its inclusion in the curriculum.

*“It is easy to introduce them when you have a necessarily local example (…) mosquito vectors… it is not bad for starting a discussion”* (teacher 3, upper secondary school).

#### 3.2.2 New Caledonia: an integrated but evolving experience

In contrast, in New Caledonia, teachers reported a long-standing pedagogical experience related to dengue.

*“For a very long time, dengue was addressed from kindergarten onwards”* (teacher 2, New Caledonia).

*“We had educational kits, materials sent by the DASS”* (teacher 5, New Caledonia).

However, the success of the Wolbachia program is now modifying the perception of risk and reshaping teachers’ priorities:

*“There is really a very strong decline of dengue here”* (teacher 4, New Caledonia).

Leptospirosis is now identified as a priority issue:

*“We have a resurgence in leptospirosis”* (teacher 2, New Caledonia).

A clear need for updated local data was expressed:

*“I would really like to have updated data… at the territorial level”* (teacher 3, New Caledonia).

In New Caledonia, proximity to risk facilitates the pedagogical integration of these topics into teaching. Infection is socially situated and perceived as concrete. However, priorities evolve according to the health context, highlighting the importance of adaptable and contextualized educational resources.

These findings highlight a clear contrast between territories. While in mainland France vector-borne diseases are predominantly perceived as distant and emerging issues, in New Caledonia they are experienced as immediate and concrete health concerns. This difference in perceived proximity appears to be a key determinant in the extent to which these topics are integrated into teaching practices. Across both territories, secondary school teachers were more likely to link these issues to broader environmental dynamics, whereas primary school teachers emphasized emotional and pedagogical challenges.

### 3.3 Theme 3—pedagogical expectations for a One Health resource: active, progressive, and interdisciplinary materials

#### 3.3.1 Students as active learners: hands-on activities and play

Across all educational levels, teachers favored active approaches involving hands-on activities, observation, inquiry-based learning, visual supports, and collaborative tasks.

*“In primary school, we talk about inquiry-based learning”* (teacher 1, primary school).

*“The fact of seeing the development of the mosquito, that interests them much more”* (teacher 7, New Caledonia).

Play was identified as a central pedagogical lever:

*“Children learn through play”* (teacher 1, New Caledonia).

The acceptability of the topic appears to depend on its translation into concrete pedagogical formats. A traditional top-down transmissive approach was considered insufficient, while active student engagement was viewed as essential.

*“They can feel invested with a mission at home (…) avoid stagnant water… they will know why they are doing something”* (teacher 4, primary school).

Several teachers also emphasized the need for explicit progression from early primary school to upper secondary education, allowing successive exploration of knowledge about the animal, ecosystem interactions, mechanisms of infection, and subsequently immunological or molecular dimensions.

*“There really needs to be progression from CP to CM2”* (teacher 1, primary school).

#### 3.3.2 Interdisciplinarity and use of scientific data

Secondary school teachers emphasized the importance of working with quantitative data and case studies.

*“Put several curves… and then with other documents that will make the link”* (teacher 1, New Caledonia).

The possibility of linking science, geography, and mathematics was perceived as facilitating.

*“It can create a link with maths, with geography”* (teacher 3, upper secondary school).

The One Health approach was viewed as an opportunity to address the topic across different subjects. However, this interdisciplinarity needs to be explicitly supported in order to be operational. Expectations nevertheless vary according to educational level: in lower secondary school, teachers requested simplified and didactically adapted scientific data, whereas in upper secondary school, some expressed the need for documentary corpora closer to research publications, enabling analytical work and simplification appropriate to students’ level.

These expectations were largely shared across territories, indicating a strong convergence in pedagogical approaches and resource needs. However, variations were observed according to educational level, with increasing expectations for scientific depth, data analysis, and disciplinary integration from lower to upper secondary education.

### 3.4 Theme 4—implementation conditions: facilitators and barriers

#### 3.4.1 Facilitators

Strong motivation emerged when resources were perceived as immediately usable. Teachers expressed a clear preference for free, ready-to-use, clear, and immediately usable resources:

*“Free, ready-made. And created by teachers”* (teacher 5, lower secondary school).

“*Ready-made, ready-made”* (teacher 6, lower secondary school).

*“It has to be directly usable”* (teacher 4, lower secondary school).

These statements reflect a structural constraint related to workload and the multiplicity of institutional demands. As a result, adoption decisions appear to rely on the availability of resources that are readily usable and quickly accessible. A “ready-made” resource reduces perceived costs (time, preparation, uncertainty) and thus becomes a decisive facilitator for appropriation.

Explicit links with curricula were identified as a key condition for legitimacy. Several teachers emphasized the need for the resource to justify and anchor the One Health entry point within the progression of learning objectives.

*“Maybe also the link with the curriculum, that is to say in the progression… When can it be addressed, how and why each time”* (teacher 4, New Caledonia).

*“Very interested since it gave meaning to 3 parts of the curriculum”* (teacher 1, upper secondary school).

*“… what legitimacy do we have to address this topic in this sequence… what are the justifications and why is it important…”* (teacher 4, New Caledonia).

The issue is not limited to compliance with the school curriculum; teachers also refer to their professional legitimacy and to the anticipation of potential contestation. Explicit curricular alignment appears to function as both pedagogical guidance and institutional protection.

The need for concise and level-adapted resources, with graduated difficulty, was strongly emphasized. Teachers highlighted the importance of materials that are visual, not overloaded with information, and adaptable to students’ abilities:

*“… images uh not too many complicated words… so that the student does not have to think for uh 25 lines of text…”* (teacher 1, lower secondary school).

*“Yes… a graduation in the difficulty”* (teacher 2, lower secondary school).

*“Yes, too much information… too much information at once, it does not work”* (teacher 1, lower secondary school).

Teachers requested pedagogical resources adapted to students’ educational level. The One Health approach is perceived as broad and complex; therefore, its implementation requires a design that structures this complexity through progression, adjustable depth, and differentiation.

The need for a structured teacher guide to provide scientific and pedagogical reassurance was frequently highlighted. Teachers expected such a guide to include definitions, key terminology, reference points, justification, and adaptability.

“*Well, a sheet yes”* (teacher 2, primary school).

“*… master the subject before going in front of the students…*” (teacher 1, primary school).

“*… a teacher guide… scientific definitions… didactically adapted… accessible, especially for primary school teachers…*” (teacher 4, New Caledonia).

The teacher guide responds to a perceived insecurity regarding professional posture, particularly among primary school teachers without a scientific background, who may question their legitimacy in addressing health and environmental topics. It therefore constitutes a direct facilitator of implementation. Several teachers also emphasized the need for assessment tools associated with the proposed activities, such as competency tracking grids, examples of evaluation, or self-assessment modalities. The expected resource is thus not limited to a teaching sequence but includes reference points that allow learning outcomes to be formalized and objectified.

The importance of visual and diverse materials to support inclusion, comprehension, and engagement was explicitly emphasized. Teachers highlighted the need for a variety of formats enabling rapid access to meaning, including for students with specific learning needs.

“*… having visuals… Dys or not Dys… UPE2A… all these images already…*” (teacher 1, lower secondary school) (NB: “Dys” refers to students with dyslexia, dyspraxia, or dyscalculia; UPE2A refers to classes for newly arrived allophone students).

“*… graphs, illustrations… photos, drawings… a really different format”* (teacher 1, lower secondary school).

Visual supports were described as pedagogical tools in their own right. They facilitate access to content for students at different levels and with diverse needs, and support active engagement in learning processes (observing, comparing, inferring).

#### 3.4.2 Barriers

Lack of time emerged as the most frequently reported barrier across all educational levels and territorial contexts, and was identified as a central organizational constraint shaping the integration of new topics into teaching.

“*It is time… because we cannot teach everything”* (teacher 3, New Caledonia).

“*I will say it again, we cannot teach everything… And then, I would just say time”* (teacher 3, New Caledonia).

Primary school teachers, who are responsible for multiple subjects, emphasized the density of curricula and the constant need to arbitrate between disciplinary priorities.

“*As such, diseases are not part of the primary school curriculum”* (teacher 1, primary school).

In lower secondary school as well, the time required to adapt teaching materials was identified as a significant constraint.

“*When we take a graph, we have to go to medical websites and we have to redo them all ourselves”* (teacher 1, lower secondary school).

“*It is rarely easy to find updated graphs… reworking them so that they are understandable for students is sometimes an immense amount of time lost for a few minutes of activity”* (teacher 3, lower secondary school).

The reported lack of time reflects heavily loaded curricula, the need to prioritize among competing topics, and the demand for immediately usable resources. The barriers identified do not stem from opposition to teaching the One Health concept, but rather from structural and professional constraints.

Limited knowledge and a perceived lack of legitimacy to teach health-related topics emerged as a significant barrier, particularly among non-specialist teachers. Limited familiarity with the One Health concept and with certain associated scientific notions contributed to this difficulty:

“*It is a notion that is new that we have not necessarily heard about… and there is not necessarily any training either”* (teacher 4, lower secondary school).

“*I would already have a big need for an explanation of the notion… that I do not master at all… of One Health”* (teacher 5, lower secondary school).

Teachers expressed the need to master the content before teaching it.

“*… master the subject before going in front of the students…”* (teacher 1, primary school).

“*… what can slow us down is the lack of knowledge*” (teacher 3, New Caledonia).

“*… lack of legitimacy to teach health education… fear…*” (teacher 2, New Caledonia).

In upper secondary school, difficulty was also associated with students’ understanding of the interconnected nature of these phenomena.

“*They do not understand that ultimately everything is linked… the climate, biodiversity, the human”* (teacher 4, upper secondary school).

Some teachers also referred to changes in initial teacher training.

“*Young teachers who did not have as much scientific education… they really need it”* (teacher 2, upper secondary school).

The barrier identified is therefore twofold: scientific, through lack of knowledge and training, and professional-identity-related, concerning teachers’ perceived legitimacy to address health topics. Teachers expressed the need for a structured and well-referenced teacher guide to support the introduction of this new concept.

The risk of anxiety among students emerged as a barrier specific to primary education. Teachers emphasized the need to avoid anxiety-inducing effects when addressing health and environmental topics. The issue does not concern reluctance to teach these subjects, but rather how they are introduced in the classroom:

“*We really need to work on this anxiety-inducing aspect. And then on top of that they say nonsense, they used that too, it turned a bit into discrimination: Ah yes, you have mosquito bites, aha ah I am avoiding you so …”* (teacher 1, primary school).

Teachers described situations in which certain content triggered strong emotional reactions among students.

“*I will give an example just by talking about whales that are endangered, et cetera. That we hunted whales to make makeup, toothbrushes and everything. So I had students who no longer wanted to eat meat, who no longer wanted uh to buy toothbrushes, et cetera … So it is true that it is sometimes quite anxiety-inducing for all the students”* (teacher 2, primary school).

Similarly, some teachers mentioned repercussions within families when students modified their behaviors following classroom teaching.

“*A teacher who had worked well on bananas and there you go, realizing the carbon impact of bananas. And well, there were children who there you go, refused to eat bananas, afterwards it is… there you go, and some parents were not very happy either (laughter). So it is true that there you go there is an approach, uh let us say… Well thought-out to have on this kind of topic”* (teacher 1, primary school).

Teachers therefore stressed the need for a pedagogical approach centered on prevention, solutions, and concrete actions, in order to avoid leaving students with an exclusively problem-focused or alarmist representation of health issues.

“*We must not remain on there are problems, we absolutely have to propose solutions”* (teacher 1, primary school).

This barrier appears specific to primary education, where the management of students’ emotions and relationships with families are described as determining factors in the choice of topics and in how they are addressed.

The lack of adapted tools and material constraints was also identified as a significant barrier. Teachers reported difficulties in finding pedagogical resources suited to their specific classroom contexts:

“*I had searched, I had not found much… found nothing adapted”* (teacher 4, primary school).

“*We find resources but they are adapted for older students”* (teacher 4, primary school).

In lower secondary school, format emerged as a central issue.

“*Too much information at once, it does not work”* (teacher 1, lower secondary school).

“*I was looking for a kind of small card format… something that the kids could have on their desk… and there was nothing”* (teacher 5, lower secondary school).

Material constraints were also mentioned.

“*I am in the very depths of the Aube… I still have chalkboards… I cannot project and write at the same time”* (teacher 5, lower secondary school).

Student heterogeneity was described as a determining factor.

“*Dys-format materials… for those who have difficulty reading”* (teacher 5, lower secondary school).

“*Something quite visual… these students are struggling because we do not have much to offer them”* (teacher 6, lower secondary school) (referring to their lower secondary school in a socioeconomically disadvantaged area).

These elements show that the barrier does not concern only scientific content, but also the format, accessibility, and adaptability of resources.

Overall, facilitators and barriers were largely consistent across territories, although some differences emerged depending on educational level and local context. While teachers in both mainland France and New Caledonia emphasized the importance of ready-to-use, structured, and adaptable resources, the perceived urgency and relevance of these topics appeared stronger in New Caledonia, where health risks are more directly experienced. Across educational levels, primary school teachers particularly highlighted issues related to time constraints, emotional impact on students, and the need for accessible and reassuring materials, whereas secondary school teachers placed greater emphasis on scientific content, data use, and interdisciplinarity.

### 3.5 Cross-cutting synthesis

#### 3.5.1 Similarities

Despite the diversity of educational levels and territorial contexts, strong convergences were observed across all focus groups. Teachers expressed a strong need for contextualization, favoring situations close to students’ lived experiences and supported by up-to-date data, ideally at the territorial level. They valued active pedagogical approaches centered on hands-on activities, inquiry, and play, in which students assume an active role. They requested structured and reassuring resources (teacher guides, definitions, scientific validation), incorporating progression adapted to different educational levels. This expectation was particularly salient among primary school teachers, but was also observed across all educational levels. Finally, time constraints constitute a major structuring parameter in decisions regarding curricular content. This constraint was consistently reported across both territorial contexts and all educational levels, highlighting its cross-cutting nature.

#### 3.5.2 Differences

The main difference lies in the degree of exposure to risk. In mainland France, vector-borne infections are predominantly perceived as emerging or indirect issues, often addressed through the lens of climate change. In New Caledonia, pedagogical experience is longer-standing and directly linked to local health concerns (dengue, leptospirosis), facilitating a more concrete and contextualized integration of these topics into teaching. Beyond territorial differences, variations were also observed across educational levels. Primary school teachers emphasized accessibility, emotional considerations (particularly the risk of anxiety), and the need for simple and concrete approaches. In contrast, secondary school teachers highlighted the importance of scientific content, the use of data, and opportunities for interdisciplinary learning. These findings suggest that while the One Health approach is broadly acceptable across contexts, its implementation requires adaptation to both territorial and educational specificities.

## 4 Discussion

This qualitative study aimed to analyze teachers’ perceptions and pedagogical needs regarding the integration of the “One Health” approach into school curricula. Four main themes emerged: limited familiarity with the One Health terminology despite an intuitive understanding of interdependencies; the importance of local context and perceived proximity to health risks; strong expectations for active, progressive, and interdisciplinary educational resources; and implementation constraints related to time, perceived professional legitimacy, management of students’ emotions, and material conditions.

### 4.1 One Health: an explicitly unfamiliar concept, yet implicitly present in reasoning

A central finding is the gap between limited familiarity with the term “One Health” and teachers’ ability to articulate connections between environmental changes, animal health, and human health. This suggests that the principles of the One Health approach are already partially embedded in educational practices through related fields such as education for sustainable development, climate education, and biodiversity topics, albeit without explicit conceptual framing. Making this framework explicit could enhance curricular coherence and support structured progression across educational stages. From a health literacy perspective (14), formalizing these interconnections may contribute to strengthening students’ understanding of the multiple determinants of health. However, the current lack of shared terminology and dedicated pedagogical tools limits the translation of these intuitive reasonings into structured and assessable learning objectives.

### 4.2 Perceived relevance depends on epidemiological and territorial context

Proximity to health risks emerged as a major determinant of teachers’ engagement. In mainland France, vector-borne diseases are often perceived as emerging or geographically distant, which may limit their integration into already densely structured curricula. This finding aligns with research on risk perception, showing that direct experience and geographical proximity strongly influence the prioritization of issues (24).

In contrast, in New Caledonia, the historical experience of dengue and the resurgence of leptospirosis facilitate a more concrete appropriation of these topics. However, changes in the epidemiological context also reshape educational priorities, highlighting the need for adaptable and context-sensitive resources. These findings support the relevance of a modular resource model incorporating updated data and locally grounded examples. They are consistent with the Health Promoting Schools framework advocated by the World Health Organization, which emphasizes that school-based interventions are more effective when anchored in local social and health realities (15).

### 4.3 Converging expectations toward practical resources compatible with professional constraints

Regardless of territory or educational level, teachers expressed a strong demand for “ready-made,” visual, adaptable, and immediately usable resources. These expectations reflect a pragmatic response to time constraints and densely structured curricula. Research in implementation science shows that ease of use and alignment with institutional constraints are major determinants of the diffusion and sustainability of innovations (25, 26).

Teachers also valued active pedagogical approaches (hands-on activities, inquiry, play), suggesting that the acceptability of the One Health approach largely depends on its translation into concrete classroom practices. Requests related to inclusivity (visual materials, differentiation, adaptation to limited equipment) further indicate that barriers are not solely conceptual but also material and organizational. Accessibility of educational resources thus constitutes a structural condition for reducing health-related educational inequalities.

### 4.4 Professional legitimacy and management of emotional dimensions

The barriers identified primarily relate to feasibility and perceived competence. Lack of time emerged as the most frequently cited cross-cutting constraint. In addition, particularly in primary education and among non-specialist teachers, a sense of scientific insecurity and lack of legitimacy to address health-related topics was reported. This dimension echoes research on teacher professional identity, which highlights the role of perceived disciplinary competence in pedagogical engagement (27).

In primary education in particular, concern about potential anxiety-inducing effects on students appeared as a specific issue. Teachers emphasized the need for a solution-oriented approach focused on preventive actions in order to avoid framing health issues exclusively in problem-based or alarmist terms. This concern aligns with health promotion models centered on empowerment, which aim to combine risk awareness with the development of action-oriented capacities (14).

### 4.5 Implications for the development of One Health educational resources within e-bug

These findings suggest several operational directions for resource design. First, explicitly articulating the One Health framework and clearly aligning it with school curricula may strengthen both legitimacy and curricular coherence. Second, developing modular, progressive, and context-sensitive resources would facilitate their appropriation across diverse territorial contexts. Third, complementing student activities with a structured teacher guide—including a solid scientific foundation, pedagogical reference points, and assessment tools—would help secure teachers’ professional posture. Within the methodological framework of e-Bug, these elements align with a co-construction approach and adaptation to real-world classroom constraints, conditions identified as crucial for the sustainability of educational interventions (25).

### 4.6 Strengths and limitations

A major strength of this study lies in the inclusion of teachers from different educational levels and from two contrasting French territories, geographically distant from one another, enabling comparison of how contextual factors influence perceived relevance and pedagogical needs. The achievement of thematic saturation further strengthens the robustness of the qualitative findings. In addition, the involvement of institutional partners in the recruitment process facilitated access to a diverse range of participants across territories and educational settings. However, differences in focus group composition between mainland France and New Caledonia, including the use of mixed educational levels and the inclusion of pedagogical advisors and an education inspector in the latter, may have introduced variations in perspectives and degrees of proximity to classroom practices, thereby limiting the direct comparability of findings between groups. Furthermore, recruitment through professional networks may have favored the participation of teachers already sensitized to health or science-related topics. The videoconference format may also have influenced participation dynamics and the depth of interaction within focus groups. Finally, the findings reflect self-reported perceptions and declared practices rather than direct observation of classroom implementation.

### 4.7 Perspectives

The focus groups should therefore not be viewed merely as a descriptive exploratory step, but as a critical phase in identifying real-world implementation conditions, enabling anticipation of barriers and the design of resources directly aligned with professional constraints.

The findings of this study directly inform the resource development phase within the e-Bug framework. Further work will involve piloting and refining One Health educational resources through classroom-based implementation studies, assessing feasibility, acceptability, and learning outcomes across educational levels. It would also be valuable to examine whether explicitly reframing existing activities through the One Health framework enhances curricular coherence, student engagement, and selected preventive behaviors. In the longer term, this work contributes to documenting a transferable model for the educational operationalization of the One Health concept, integrating updated scientific evidence, alignment with school curricula, co-construction with field stakeholders, and institutional support. These findings contribute to the growing body of literature seeking to operationalize One Health beyond biomedical and policy frameworks and into educational systems.

## 5 Conclusion

This qualitative study represents a pre-implementation exploratory phase, essential within models of educational innovation diffusion. It highlights that, despite limited familiarity with the “One Health” terminology, teachers already mobilize systemic reasoning linking human, animal, and environmental health. However, the operational integration of One Health in schools appears contingent upon concrete determinants: territorial proximity to health risks, availability of localized data, time constraints, perceived lack of scientific legitimacy, management of anxiety in primary education, and material barriers. Our findings underscore the value of ready-to-use, progressive, interdisciplinary, and adaptable educational resources, combined with a structured and scientifically validated teacher guide. Within the methodological framework of e-Bug, these elements constitute directly actionable levers for designing and disseminating One Health resources aligned with real-world classroom constraints and potentially transferable across diverse socio-territorial contexts. The next steps will involve the co-construction and classroom-based piloting of these resources, followed by evaluation of their feasibility, acceptability, and impact on learning outcomes.

## Data Availability

The raw data supporting the conclusions of this article will be made available by the authors, without undue reservation.
