# Supplementary material for: Integrating the One Health approach into school curricula: perceptions and educational needs of teachers in mainland France and New Caledonia
Source: Front Public Health. 2026 Apr 24;14:1822865. doi: 10.3389/fpubh.2026.1822865 (PMC13153090; doi:10.3389/fpubh.2026.1822865)
Supplement: Supplementary file 1 [file Table_1.docx]

## Interview guide for focus groups on the development of educational resources on a One Health framework

**Introduction and consent**

Hello, my name is …, and I work at the … University Hospital. Our department aims to improve population health and well-being. We coordinate the e-Bug programme, an international partnership that develops educational resources for schools and communities to teach children about infections, including their transmission, prevention, and treatment. These resources mainly consist of lesson plans adapted to different educational levels, from primary to upper secondary school.

We are currently developing new resources based on the One Health approach. The One Health approach recognizes that the health of humans, animals (both domestic and wild), plants, and the environment are closely interconnected and interdependent.

For example, vector-borne diseases are transmitted by vectors such as insects, which can carry harmful microbes between animals and humans or among humans. These diseases are responsible for more than 700,000 deaths worldwide each year. In recent years, vectors have expanded into new regions, increasing the risk of transmission and infection. It is estimated that around 80% of the global population is now at risk of one or more vector-borne diseases. This evolution is linked to climate change, international travel, and the adaptation of vectors to new environments.

Promoting awareness and understanding of the One Health approach is therefore essential to protect population health. Educating children is particularly important, as they can act as messengers within their families and communities.

Today, we will ask you questions about your knowledge and experiences related to vector-borne infections and the One Health approach. This is not a test of knowledge, but an opportunity to understand your perspectives and needs for developing educational resources.

Before we begin:

- The discussion will last approximately 60 minutes.

- There are no right or wrong answers; we are interested in your experiences and opinions.

- Please respect each other’s viewpoints and allow everyone to speak.

- The discussion will be audio-recorded to ensure accurate transcription. The recording will be used only for research purposes and will remain confidential.

- You may withdraw at any time without any consequences.

- All data will be anonymized, and any quotations used will not be identifiable.

Do you agree to participate and to the recording of this discussion?

**Introductions**

To begin, please briefly introduce yourself (name, role, educational level, and subject taught).

Knowledge and perceptions

1. What does the One Health approach mean to you?
2. In recent years, links have been established between climate change and vector-borne diseases. Are you aware of this, and what are your thoughts?

Teaching practices

1. Do you think the One Health approach should be taught in schools?

At what age?

In which subjects?

1. Are you aware of existing educational resources on this topic?
   If yes :
   For which age groups?

What aspects of the topic are covered?

What has been your experience teaching this topic?

Were students engaged and interested?

What did they understand or find difficult?

1. How could this topic be integrated into the curriculum?

In which subjects (e.g., biology, geography)?

Have you already included it?

Where would you consider including it?

Current resources

1. If you have already taught this topic, please describe the lesson.
   Did you use resources? If yes, where did you find them?

What types of activities were included?

How long did the lesson last?

1. Where would you look for resources on this topic?
2. What do you like or dislike about the resources you currently use?

Motivation and perceived importance

1. What would encourage you to teach this topic?
   For example, if it is not explicitly included in the national curriculum?
2. How important do you think it is to teach this topic to students?
   What impact do you expect?

What learning objectives would you set?

1. What would motivate you to use a new resource?

Needs for new resources

1. What type of resources would be useful on this topic?
2. What was missing from previous resources?
3. What key concepts should be included in the lesson content? (Age-specific needs)
4. What types of activities would you like to see?
5. What should be included in the teacher guide? How much information would be appropriate?
6. What challenges do you foresee in teaching One Health concepts?
7. Which vector-borne diseases in a One Health approach would be relevant to include?
8. What duration would be appropriate? A single lesson or a sequence of lessons?

Training and support

1. What type of training or support would you need to feel comfortable teaching the One Health approach?
2. What should it include?

Barriers and facilitators

1. What barriers do you foresee in using such resources in schools?
   How could these barriers be overcome?
2. How could this resource be promoted among teachers?

Is there anything else you would like to add regarding this topic or the use of such resources in your teaching?

Thank you for your participation.
